# Influence of the* Melissa officinalis* Leaf Extract on Long-Term Memory in Scopolamine Animal Model with Assessment of Mechanism of Action

**DOI:** 10.1155/2016/9729818

**Published:** 2016-04-28

**Authors:** Marcin Ozarowski, Przemyslaw L. Mikolajczak, Anna Piasecka, Piotr Kachlicki, Radoslaw Kujawski, Anna Bogacz, Joanna Bartkowiak-Wieczorek, Michal Szulc, Ewa Kaminska, Malgorzata Kujawska, Jadwiga Jodynis-Liebert, Agnieszka Gryszczynska, Bogna Opala, Zdzislaw Lowicki, Agnieszka Seremak-Mrozikiewicz, Boguslaw Czerny

**Affiliations:** ^1^Department of Pharmaceutical Botany and Plant Biotechnology, Poznan University of Medical Sciences, Sw. Marii Magdaleny 14, 61-861 Poznan, Poland; ^2^Department of Pharmacology and Phytochemistry, Institute of Natural Fibres and Medicinal Plants, Wojska Polskiego 71b, 60-630 Poznan, Poland; ^3^Department of Pharmacology, University of Medical Sciences, Rokietnicka 5a, 60-806 Poznan, Poland; ^4^Department of Pathogen Genetics and Plant Resistance, Metabolomics Team, Institute of Plant Genetics of the Polish Academy of Science, Strzeszynska 34, 60-479 Poznan, Poland; ^5^Laboratory of Experimental Pharmacogenetics, Department of Clinical Pharmacy and Biopharmacy, University of Medical Sciences, 14 Sw. Marii Magdaleny, 61-861 Poznan, Poland; ^6^Department of Stem Cells and Regenerative Medicine, Institute of Natural Fibres and Medicinal Plants, Wojska Polskiego 71b, 60-630 Poznan, Poland; ^7^Department of Toxicology, Poznan University of Medical Sciences, Dojazd 30, 60-631 Poznan, Poland; ^8^Division of Perinatology and Women's Diseases, Poznan University of Medical Sciences, Polna 33, 60-535 Poznan, Poland; ^9^Laboratory of Molecular Biology, Poznan University of Medical Sciences, Polna 33, 60-535 Poznan, Poland; ^10^Department of General Pharmacology and Pharmacoeconomics, Pomeranian Medical University, Zolnierska 48, 70-204 Szczecin, Poland

## Abstract

*Melissa officinalis* (*MO*, English: lemon balm, Lamiaceae), one of the oldest and still most popular aromatic medicinal plants, is used in phytomedicine for the prevention and treatment of nervous disturbances. The aim of our study was to assess the effect of subchronic (28-fold) administration of a 50% ethanol extract of* MO* leaves (200 mg/kg, p.o.) compared with rosmarinic acid (RA, 10 mg/kg, p.o.) and huperzine A (HU, 0.5 mg/kg, p.o.) on behavioral and cognitive responses in scopolamine-induced rats. The results were linked with acetylcholinesterase (AChE), butyrylcholinesterase (BuChE), and beta-secretase (BACE-1) mRNA levels and AChE and BuChE activities in the hippocampus and frontal cortex of rats. In our study,* MO* and HU, but not RA, showed an improvement in long-term memory. The results were in line with mRNA levels, since* MO* produced a decrease of AChE mRNA level by 52% in the cortex and caused a strong significant inhibition of BACE1 mRNA transcription (64% in the frontal cortex; 50% in the hippocampus). However, the extract produced only an insignificant inhibition of AChE activity in the frontal cortex. The mechanisms of* MO* action are probably more complicated, since its role as a modulator of beta-secretase activity should be taken into consideration.

## 1. Introduction

Neurodegenerative disorders including Alzheimer's disease, characterized by loss of memory and learning ability, are the increasing public health problem worldwide [1, 2]. Plants with neurobiological activity may be potential targets for drug discovery [3]. Searching for new drugs and explaining their mechanisms of action are one of the most intensively developing areas of scientific platform. Moreover plant origin substances can be a valuable alternative way in the prevention and treatment of dementias as component of healthy diet.

*Melissa officinalis* (*MO*, English: lemon balm, Lamiaceae), one of the oldest and still most popular aromatic medicinal plants, is used in phytotherapy for the prevention and treatment of nervous disturbances of sleep and gastrointestinal disorders as sedative and antispasmodic medicine [4]. New neuropharmacological investigations showed that ethanol extracts of* MO* exerted also neuroprotective [5, 6], antioxidant, cyclooxygenase-2 inhibitory [7], and antinociceptive activities [6, 8]. Moreover, it is known that* MO* is used for memory-enhancing effects in European folk medicine [9–12]. Indeed, Akhondzadeh et al. [13] carried out the clinical trial in which* MO* extract produced a significantly better outcome on cognitive function than placebo in patients with mild to moderate Alzheimer's disease. In other clinical studies, Kennedy et al. [14–16] observed that a treatment combining both calming effects and beneficial cholinergic modulation may well prove to be a novel treatment for Alzheimer's disease. Studies of molecular mechanisms showed that* MO* extracts exhibited cholinergic (nicotinic and muscarinic) receptor-binding properties in human cerebral cortex tissue [15]. Moreover, it was observed that both fractions and crude ethanol extract of* MO* inhibited acetylcholinesterase (AChE) of rats brain [17, 18] and also* in vitro* [9, 19, 20], but only two studies analyzed behavioral mechanism of action of* MO* extracts on scopolamine-induced memory impairment in rats [12, 18]. One study showed that* MO* extract (after intraperitoneal injection) significantly enhanced learning and memory of rats and significantly ameliorated scopolamine-induced learning deficit [18]; however in another study, it was observed that* MO* extract was completely inactive [12]. Moreover, in these studies, attention has not been paid to the influence of the* MO* extract on the expression of genes participating in the conditioning of the synaptic cholinergic equilibrium, AChE and bytyrylcholinesterase (BuChE) or even the beta-secretase (BACE1), in rats brain, being responsible for beta-amyloid deposition in Alzheimer's disease [1].

On the other hand, it is well known that essential oil in leaf of* MO* is considered to be the therapeutic principle mainly responsible for most of the abovementioned activities, but also plant phenolics are considered as an important factor in* MO* therapeutic effects [6, 21]. It was shown that ethanol extract contains rosmarinic acid (RA) as the major compound [7]; however there is no detailed information about full phenolic profile of extract from* MO* leaves being probably responsible for pharmacological effects as well. This becomes especially important given the results of previous studies showing that RA did not affect short- and long-term memory [22] or marginally improved long-term memory in rats model [23], although it was observed that RA had an ability to inhibit AChE in the frontal cortex and in the hippocampus of rats [23]. On the other hand, polyphenols still constitute a promising source of new drugs and there is a high interest in understanding their mechanisms in prevention and treatment of Alzheimer's disease [16, 23, 24].

## 2. Objectives

The aim of this study was to evaluate the influence of subchronic (28-fold) intragastrical administration of ethanol extract of* MO* leaves and rosmarinic acid on scopolamine (SC) impaired memory in animal model. Furthermore, acetylcholinesterase (AChE) and butyrylcholinesterase (BuChE) activities assessment in hippocampus and frontal cortex were studied. Moreover, gene expression levels for AChE, BuChE, and BACE-1 in the hippocampus and frontal cortex were investigated. The* MO* ethanolic extract was phytochemically investigated (HPLC-ESI-MS^n^, UPLC-PDA) in order to identify phytochemicals present in plant extract.

## 3. Materials and Methods

### 3.1. Plant Material

The leaves of* Melissa officinalis* L. (Lamiaceae) were obtained from an herbal company “Kawon-Hurt” (Gostyn Wlkp., Poland). The plant material was identified in the Department of Pharmaceutical Botany and Plant Biotechnology, Faculty of Pharmacy, Poznan University of Medical Sciences. The voucher specimen has been deposited in the Herbarium of the Institute of Natural Fibres and Medicinal Plants in Poznan (Plewiska), Poland.

### 3.2. Chemical and Drugs

All reagents for HPLC analysis, scopolamine hydrobromide trihydrate (SC), and rosmarinic acid (RA) and reagents for biochemical analyses were purchased from Sigma-Aldrich (Poland). Huperzine A (HU) was obtained from Enzo Life Sciences AG (Alexis Corporation, Biomibo Distribution, Poland). Chemicals for gene expression analysis were obtained from Roche Diagnostic and ALAB (Poland). All chemicals and drugs were* ex tempore* prepared on the day of the experiment.

### 3.3. Preparation of the Extract

1000 g of raw plant material was extracted with 50% ethanol by percolation (24 h) at room temperature (22 ± 1°C). After filtration, the extract was concentrated under vacuum to eliminate the ethanol content. The concentrated extract was frozen and freeze-dried. The final product yielded 248.21 g of solid extract.

### 3.4. Metabolites Identification with LC-MS

Metabolomic analyses were performed using two complementary LC-MS systems. The first one, HPLC-DAD-MS^n^, consisted of an Agilent 1100 HPLC instrument with a diode-array detector (DAD) (Agilent, Palo Alto, CA, USA) and an Esquire 3000 ion trap mass spectrometer (Bruker Daltonics, Bremen, Germany). Chromatographic separations by HPLC were carried out on an XBridge C18 column (150 × 2,1 mm, 3,5 *μ*m particle size) using water acidified with 0.1% formic acid (solvent A) and acetonitrile (solvent B) with the mobile phase flow of 0.2 mL/min in the following gradient: 0–25 min from 10% to 30% B, 25–46 min to 98% B, and being maintained at these conditions until 51 min. Up to 52 min system returned to the starting conditions and was reequilibrated for 5 min. The most important MS parameters were as follows: the ion source ESI voltage −4 kV or 4 kV, nebulization of nitrogen at a pressure of 30 psi at a gas flow rate 9 L/min, ion source temperature at 310°C, and skimmer 1: −10 V. The spectra were scanned in the range of 50–3000 *m*/*z*. The second system consisted of UPLC (the Acquity system, Waters, Milford, USA) hyphenated to QExactive hybrid MS/MS quadrupole-Orbitrap mass spectrometer. Chromatographic separations in this system were carried out using water acidified with 0.1% formic acid (solvent A) and acetonitrile (solvent B) with the mobile phase flow of 0.4 mL/min in the following gradient: 0–5 min from 10% to 25% B, 5–13 min to 98% B, and being maintained at these conditions until 14.5 min. Up to 15 min system returned to the starting conditions and was reequilibrated for 3 min. QExactive MS operated upon the following settings: the HESI ion source voltage −3 kV or 3 kV. The sheath gas flow was 48 L/min, auxiliary gas flow 13 L/min, ion source capillary temperature 250°C, and auxiliary gas heater temperature 380°C. The CID MS/MS experiments were performed using collision energy of 15 eV. The MS^n^ (up to the MS5) and MS/MS spectra were recorded in the negative and positive ion modes using the previously published approach [23, 25, 26]. The individual compounds were identified via comparison of the exact molecular masses (measured in most cases with Δ below 1 ppm), mass spectra, and retention times to those of the standard compounds, as well as the databases available online (PubChem, ChEBI, Metlin, and KNApSAck) and literature data.

### 3.5. Quantitative HPLC Analysis

Sample was extracted by 70% ethanol. After sonification, solution was cooled down and filtered through membrane filter. HPLC method was used to determine RA in a dry ethanolic extract. The Lichrospher 100 RP-18e (125 mm; 4,0 mm; 5 um, Merck) was applied for identification of this active compound. Temperature of column was 35°C, detection of RA was at 205 nm, and flow rate was 1,5 mL/min. Mobile phase A was H_3_PO_4_ : H_2_O (1 : 999); mobile phase B was acetonitrile. Time was as follows: 0 min, 10% B; 13 min, 22% B; 14 min, 40% B; 25 min, 40% B.

Moreover, for identification of other chemical compounds, a Zorbax Poroshell 120 SB-C18 column, 2.7 mm 3.0 mm × 100 mm (Agilent), was used. The lithospermic acid and salvianolic acid B were detected at 250 nm; salvianolic acid A was detected at 280 nm. The gradient mixtures of phase A—water : H_3_PO_4_ (100 : 0.02, V/V)—and of phase B—acetonitrile : tetrahydrofurane (100 : 2, V/V)—were used as eluents. Peaks were identified by addition of standard solutions and by UV-Vis spectra. The quantification of these compounds was achieved using calibration curves prepared with pure compounds. The flow rate was 0.7 mL/min, column temperature 27°C, and sample injection 5 mL. The gradient mixtures program was as follows: 0 min: 5% B; 3 min: 10% B; 5 min: 12% B; 11 min: 21.7% B; 15 min: 39% B; 39 min: 39% B; 70 min: 70% B; and detection of compounds took place at 250 nm, 280 nm, and 330 nm [27].

### 3.6. Determination of Total Phenolic Compounds in the Extract

The calculation of polyphenols to gallic acid was done using the Folin-Ciocalteu reagent with the spectrophotometric modified method described by Slinkart and Singleton [28].

### 3.7. Determination of Total Hydroxycinnamic Acid Derivatives

Determination of total hydroxycinnamic acid derivatives calculated on rosmarinic acid was performed according to the procedure described in EurPh. 5.0.

### 3.8. Distillation of Essential Oil

The essential oil contents were determined by way of stream distillation in Deryng's apparatus according to EurPh. 5.0. 100.0 g of the dry hydroethanolic* MO* leaf extract (separate sample) was placed in a round-bottom flask. Then, 500.0 mL distilled water and 0.3 mL xylen were added and boiled in Deryng's apparatus for 3 h.

### 3.9. Gas Chromatography Analysis

Gas chromatography (GC) analyses were carried out using a Perkin-Elmer Clarus 500 gas chromatograph with a data processing system and an FID (GC-FID). Separation was achieved by using an Elite FFAP fused-silica capillary column (30 m long, 0.32 mm in internal diameter, and 0.25 *μ*m of film thickness). The injector and detector temperatures were 220°C. Helium was used as a carrier gas with a flow of 1.5 mL min^−1^. A sample of 1.0 *μ*L was injected, using slit mode (split ratio 1 : 100). The results were reported as the relative percentage of the total peak area.

### 3.10. Animals

Experiments with rats were performed in accordance with Polish governmental regulations (Dz. U. 05.33.289). The study was conducted in accordance with ethical guidelines for investigations in conscious animals and the study protocol was approved by the Local Ethics Committee of the Use of Laboratory Animals in Poznan, Poland (64/2008). The experiments were performed on male six-week-old Wistar rats housed in controlled room temperature (20 ± 0.2 C) and humidity (65–75%) under a 12 h : 12 h light-dark cycle (lights on at 7 a.m.). Animals were kept in groups in amounts of 8–10 in light plastic cages (60 × 40 × 40 cm) and had a free access to standard laboratory diet (pellets-Labofeed B) and to tap water in their cages.

### 3.11. Treatments

The rats were treated with hydroethanolic extract of* Melissa officinalis* leaf (*MO*) in a dose of 200 mg/kg b.w., intragastrically (p.o.) (groups* MO* + H_2_O and* MO* + SC) for 28 (28x) consecutive days. For comparative purposes, huperzine A (HU) was administered chronically (28x) in a dose 0.5 mg/kg b.w. (p.o.) (groups HU + H_2_O and HU + SC) as a known acetylcholinesterase inhibitor. Moreover, rosmarinic acid (Sigma-Aldrich) (RA) was applied (28x) in a dose of 10 mg/kg b.w. (p.o.) (groups RA + H_2_O and RA + SC) as a comparative chemical compound. On the last day, 30 min after the last dose of* MO*, or HU, SC was given intraperitoneally (i.p.) in a dose of 0.5 mg/kg b.w. Control groups were treated with 0.5% methylcellulose (MC), whereas water for injection (H_2_O) was used as a vehicle for SC (groups MC + H_2_O and MC + SC).* MO* was prepared ex tempore before administration and suspended in MC in concentrations of 20 mg/mL. On day 28 of the experiment, 1 h after the last dose, the animals were killed by decapitation and hippocampus and part of frontal cortex were collected from brain of rats. The tissue samples were then stored at −80°C until measurement of acetylcholinesterase (AChE) and butyrylcholinesterase (BuChE) activities or mRNA level changes.

### 3.12. Cognitive and Behavioral Tests

Cognitive and behavioral tests were used in the present study similarly as in our previous report [23]: (1) sedative activity was assessed using a locomotor activity test, (2) motor coordination assessment was done using a “chimney” test, (3) the passive avoidance test was performed as an animal model for the assessment of long-term memory, and (4) the object recognition test was used as an animal model for the assessment of short-term memory.

#### 3.12.1. Measurement of Locomotor Activity

Locomotor activity assessment was performed with licensed activity meter (Activity Cage, Ugo Basile, Italy) by placing the animals in the centre of the apparatus and recording their horizontal activity [23]. The data obtained were expressed as signals corresponding to animal movements for 5 minutes. The locomotor activity was measured 30 minutes after the administration of a single dose of SC H_2_O. Any distracting factors were reduced to the minimum (noise, presence of people, and presence of other rats).

#### 3.12.2. Measurement of Motor Coordination

Motor coordination was evaluated using “chimney” test described originally for mice [29]. Thirty minutes after SC or vehicle injection, rat was allowed to enter a glass laboratory cylinder that is 500 mm long and 80 mm in diameter laid on its side. Upon reaching its bottom by the animal, position of the cylinder was rapidly changed from horizontal to vertical and a timer started. The animal immediately began to move backwards. The timer was stopped after the rat left the cylinder and assumed a sitting posture on the top of the vessel. The time of exit from the cylinder was accepted as a measure of motor coordination. Motor impairment was assessed as the inability of rats to climb backwards up the tube within 60 s.

#### 3.12.3. Passive Avoidance Test

Passive avoidance test was used as an animal model for the assessment of long-term memory (effects on retrieval and memory consolidation) [30]. The test relies on the natural preference of rats for darkness. After 2 minutes of habituation to a dark compartment, a rat was placed on the illuminated platform and allowed to enter the dark compartment using licensed apparatus (Passive Avoidance System, step through, Ugo Basile, Italy). Two more approach trials were allowed on the following day with a two-minute interval between them. At the end of the second trial, an unavoidable scrambled electric footshock (500 *μ*A, AC, 3 s) was delivered through the grid floor of the dark compartment (learning trial). Retention of the passive avoidance response (latency) was tested 24 h later by placing the animal on the platform and measuring the latency in reentering the dark compartment against the arbitrary maximum time of 180 s. The test was performed after 30 minutes after the administration of a single dose of SC or the vehicle.

#### 3.12.4. Object Recognition Test

Object recognition test was used as an animal model for the assessment of short-term memory [31]. The object recognition task took place in a 40 × 60 cm open box surrounded by 40 cm high walls made of plywood with a frontal glass wall. All animals were submitted to a habituation session where they were allowed to freely explore the open field for 5 min. No objects were placed in the box during the habituation trial. On the day of testing, the animals were given an additional 3 min rehabituation period prior to commencing the test. The test was divided into three phases with two trials, the acquisition trial, the retention trial, and an intertrial interval of varying times.

(i) Acquisition trial: in this first trial, the animals explored two identical objects (*A*1 and *A*2) for a period of 3 min positioned in two adjacent corners, 10 cm from the walls.
(ii) Intertrial interval (ITI): the animals were returned to the home cage for 30 min.
(iii) Retention trial: in this second trial, the animals explored a familiar object (*A* ^*∗*^) that is a duplicate of those objects from the acquisition trial (to minimize olfactory cues) and a novel object (*B*) for a further 3 min.

They were made of a biologically inert substance (plastic) and were chosen to enable ease of cleaning (10% alcohol) between subjects in an attempt to remove olfactory cues. Object exploration is defined by animals licking, sniffing, or touching the object whilst sniffing but not leaning against, turning round, or standing or sitting on the object. Objects were of sufficient weight and were secured to the floor of the arena to ensure that they could not be knocked over or moved around by the animal. The exploration time (s) of all objects was recorded via stopwatch for subsequent statistical analysis. The time measured as an exploration behavior was used to calculate a memory discrimination index (OR) as reported by Blalock et al. [31]: OR = (*B* − *A* ^*∗*^)/(*B* + *A*), where *B* was the time spent exploring the new object and *A* ^*∗*^ was the time spent exploring the familiar object. Higher OR was considered to reflect greater memory ability [31]. The test was performed after 30 minutes after the administration of a single dose of SC or the vehicle.

### 3.13. Acetylcholinesterase and Butyrylcholinesterase Activities Assay in Brain of Rats

Acetylcholinesterase (AChE) and butyrylcholinesterase (BuChE) activities were performed by modifying spectrophotometric Ellman's method according to Isomae et al. [32]. The activities of AChE and BuChE were determined by measuring the formation of the yellow anion obtained from the reaction between Ellman's reagent and the thiocholine generated by the enzymatic hydrolysis of acetylthiocholine iodide (ATCh) and butyrylthiocholine (BTCh), respectively (sample 0.1 mL, PBS 0.8 mL, DTNB 0.1 mL, ATCh 0.20 mL, and BTCh 0.20 mL). The biochemical assay of AChE and BuChE in homogenate of brain samples was expressed as *μ*mol/min/mg protein by using spectrophotometric method (*λ* = 412 nm).

### 3.14. RNA Isolation and Reverse Transcription Reaction

Total RNA isolation from the rats brain tissues homogenates (frontal cortex, hippocampus) was carried out using TriPure Isolation Reagent (Roche) according to manufacturer's protocol. The integrity of RNA was visually assessed by a conventional agarose gel electrophoresis and the concentration will be evaluated by measuring the absorbance at 260 and 280 nm in a spectrophotometer (BioPhotometer Eppendorf). RNA samples were stored at −80°C until use. The 1 *μ*g of total RNA from all samples was used for the reverse transcription into cDNA using Transcriptor First Strand Synthesis Kit (Roche) according to manufacturer's protocol. Obtained cDNA samples was stored at −20°C or used directly for the quantitative real-time PCR (qRT-PCR).

### 3.15. Real-Time PCR mRNA Quantification

The acetylcholinesterase (AChE), butyrylcholinesterase (BChE), and beta-secretase (BACE1) genes expression level was analyzed by two-step quantitative real-time PCR (qRT-PCR), in a volume of 10 *μ*L reaction mixture, using relative quantification methodology with a LightCycler TM Instrument (Roche, Germany) and a LightCycler Fast Start DNA Master SYBR Green I kit (Roche Applied Science) according to the instructions of the manufacturer. All primers sequences were designed and custom-designed using the Oligo 6.0 software (National Biosciences) and were verified by assessment of a single PCR product on agarose gel and by a single temperature dissociation peak (melting curve analysis) of each cDNA amplification product. An GAPDH gene was used as a housekeeping gene (endogenous internal standard) for normalization of qPCR. For each quantified gene, standard curves were prepared from dilution of cDNA and generated from a minimum of four data points. All quantitative PCR were repeated twice. The data were evaluated using LightCycler Run 4.5 software (Roche Applied Science). Each PCR run included a nontemplate control to detect potential contamination of reagents.

### 3.16. Statistical Analysis

All values were expressed as means ± SEM. The statistical comparison of results was carried out using one-way analysis of variance (ANOVA) followed by Duncan's* post hoc* test for detailed data analysis. The values of *p* < 0.05 were considered as a statistical significant difference.

## 4. Results

### 4.1. Phytochemical Profile of Extract

#### 4.1.1. Identification of Metabolites

**Figure 1:**
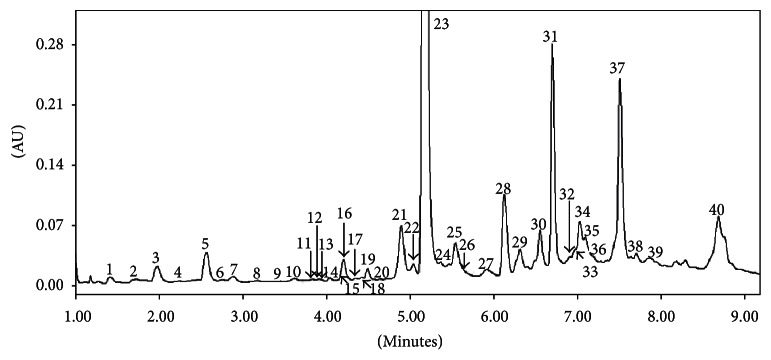
Chromatogram UV of* Melissa officinalis* leaf extract obtained at 270 nm with peaks identified by HPLC-UV-MS.

Forty phenolic metabolites were identified in hydroethanolic* Melissa officinalis* leaf extract (Table 1, Figure 1). The predominant identified compounds were bioactive caffeic acid esters and glycosides of flavones. The caffeic acid dimer, rosmarinic acid (metabolite** 23**), and caffeate trimer (lithospermic acid,** 31**) were the principal caffeic acid derivatives in the analyzed samples. Hydroxyjasmonic acid and its derivatives, teucrol as well as sagecoumarin, were identified for the first time in the genus* Melissa* while luteolin and apigenin glycoconjugates are well known phytochemicals in this species [33]. Multistep fragmentation with accurate mass measurement enabled confirming that the losses of fragment 79.9573 amu from the [M-H]^−^ ions of compounds** 4**,** 9**,** 27**,** 30**, and** 32 **referred to sulphate groups. The first-order fragmentation of** 30** revealed the loss of 79.9573 and yielded the product ion at 719.1622 *m*/*z*. The following fragmentation of this ion corresponded to that of the sagerinic acid (dimer of rosmarinic acid) described by Barros et al. [34]. The exact placement of the sulphation position would require in-depth chemical analysis. Thus,** 30** was tentatively assigned as sulphated sagerinic acid.

**Figure 2:**
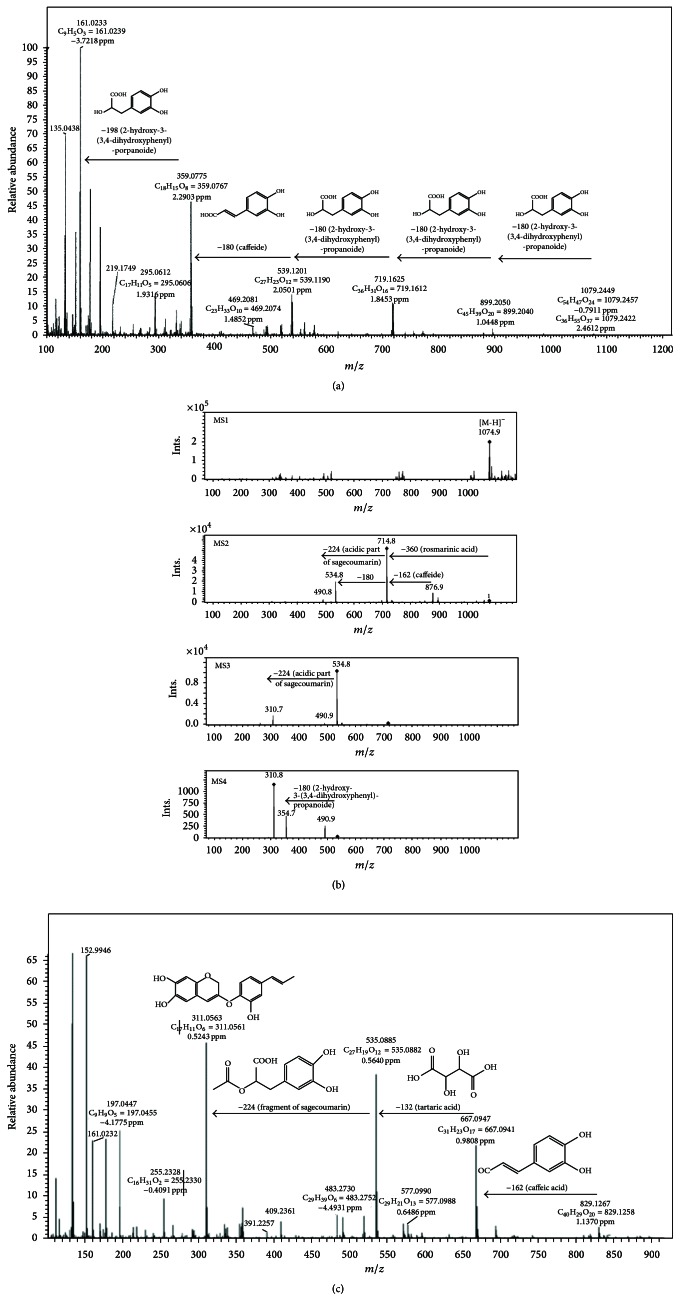
(a) Mass spectra in negative ionization mode and simplified fragmentation scheme of compound** 25** (pentameric ester of caffeic acid). (b) Mass spectra in negative ionization mode and simplified fragmentation scheme of compound** 26** (pentameric structure of sagecoumarin di-2-hydroxy-3-(3,4-dihydroxyphenyl)-propanoide caffeide). (c) Mass spectra in negative ionization mode and simplified fragmentation scheme of compound** 36** (pentameric structure of sagecoumarin caftaride).

Tetrameric structures of hydroxycinnamic acids were identified in lemon balm recently [34, 35]. The measurement of accurate masses allowed the identification of compound** 25**, which was tentatively identified as pentameric ester of caffeic acid (Figure 2(a)). In** 25**, the double loss of 180.0421 amu corresponded to fragments with the molecular formula of C_9_H_8_O_4_, adequate to dehydroxylated 2-hydroxy-3-(3,4-dihydroxyphenyl)-propanoic acid. The product ion at 719.1623 *m*/*z* and its further fragmentation are similar to those of metabolite** 22** as described previously [34, 35]. Therefore, metabolite** 25** was tentatively assigned as sagerinic acid di-2-hydroxy-3-(3,4-dihydroxyphenyl)-propanoide. Nevertheless, comprehensive studies by nuclear magnetic resonance are required to complete elucidation of substitution pattern for particular components of those pentamers.

Sagecoumarins were previously identified in* Salvia officinalis* as caffeic acid trimers [36]. Our MS analysis indicated the presence of such compounds and their derivatives also in* M. officinalis*. The pseudomolecular ion of compound** 33** observed in the negative ionization mode had the accurate mass of 535.0880 *m*/*z* which corresponded to the chemical formula C_27_H_19_O_12_ adequate for sagecoumarin (according to the Metlin and KNApSAck databases). Noteworthy,** 26**,** 33**,** 36**, and** 37** had the same fragmentation pattern of the product ion obtained in the MS/MS and MS^n^ in the negative ionization mode. The [M-H-180.0422]^−^ ion corresponded to the detachment of 2-hydroxy-3-(3,4-dihydroxyphenyl)-propanoic acid; thus** 37** was tentatively assigned as tetrameric sagecoumarin 2-hydroxy-3-(3,4-dihydroxyphenyl)-propanoide. The [M-H-360.0846]^−^ ion in** 26** was indicated on rosmarinic acid substitution to** 37**. Mass spectra of MS^n^ in negative ionization of the compound provided complementary information to HR-MS/MS mass spectra indicated on caffeic acid as internal component of the dimer. In addition, simultaneous loss of fragments 180 amu and 224 amu in MS3 and MS4 indicated that the two components cannot be linked (Figure 2(b)). However, detailed analysis of substitution pattern of** 26** should be done. Therefore,** 26** was tentatively assigned as pentameric structure of sagecoumarin di-2-hydroxy-3-(3,4-dihydroxyphenyl)-propanoide caffeide. Rupture of caffeic and tartaric acid moieties from the product ion at 535.0885 *m*/*z* was observed for compound** 36** (Figure 2(c)). Detection of the accurate masses of these two detached fragments with Δ less than 1 ppm eliminated the possibility of hexose and pentose substitution which have the same nominal masses as caffeic and tartaric acid, respectively. Therefore,** 36** was tentatively identified as another pentameric structure of sagecoumarin caftaride.

Metabolite** 28** with the accurate masses of 461.073 *m*/*z* was tentatively identified as luteolin O-glucuronide. The [M-H-176.0324]^−^ ion is indicated on loss of structure C_6_H_8_O_6_ corresponding to glucuronide moiety. The product ion at 285.0405 *m*/*z* was indicated on flavone luteolin. The place of substitution of the carboxylic acid on flavone skeleton is problematic due to different isomers reported in lemon balm: luteolin 3′-*O*- and 7*-O*-glucuronide [33]. It is impossible to distinguish both structures by mass spectrometry. Only one chromatographic peak corresponding to the [M-H]^−^ ion at 461.073 *m*/*z* was observed in our study. Since luteolin 3′-O-glucuronide was assigned as the most abundant flavonoid in lemon balm [33], we assumed that** 28** corresponded to this structure.

#### 4.1.2. Flavonoids and Polyphenolic Acids

The major compound, from the 40 chemical compounds identified in hydroethanolic* MO* leaf extract established by HPLC, was RA (8.85 g/100 g) (Table 1, Figure 1).

Other chemical compounds with neuromodulatory activities such as lithospermic acid (0.042 g/100 g), salvianolic acid A (0.040 g/100 g), salvianolic acid B (0.023 g/100 g), and caffeic acid (0.087 g/100 g) were also documented in literature. Moreover, the total polyphenolic compounds content of* MO*, determined with the use of Folin–Ciocalteu assay, was 33.97%, calculated as gallic acid. The total hydroxycinnamic derivatives content expressed spectrophotometrically as rosmarinic acid was 21.15 g/100 g.

#### 4.1.3. Essential Oil Composition

Hydroethanolic* MO* leaf extract contained 0.08% of total essential oil. The GC/FID analysis showed that the extract comprised camphene (0.04%), alfa-pinene (0.07%), beta-pinene (16.47%), and myrcene (19.51%). Moreover, according to retention time, 16 compounds were identified as follows: alfa-bisabolol, borneol, carvone, chamazulene, cineole, eugenol, gamma-terpineol, guaiazulene, isopulegol, linalool, limonene, menthol, menthyl acetate, pulegone, terpine, and thymol. These compounds are present in the essential oil in trace amounts which do not allow the quantitative interpretation.

### 4.2. Cognitive and Behavioral Experiments

#### 4.2.1. Locomotor Activity

A one-way ANOVA analysis revealed significant differences in the locomotor activity of rats expressed as their horizontal spontaneous activity after* MO* administration (ANOVA, *F*(7,80) = 6.46, *p* < 0.05) (Table 2). Detailed* post hoc* analysis showed that* MO* + H_2_O decreased the locomotor activity of rats by 40.31%, but HU + H_2_O did not affect this activity when compared with control group (MC + H_2_O). We observed also that RA + H_2_O did not change the locomotor activity of rats. Stimulating effects in the locomotor activity of rats were observed after an acute SC injection (MC + SC versus MC + H_2_O, *p* < 0.05) and this effect was observed in all SC-treated rats when compared with the proper non-SC-treated animals. On the contrary, these SC-treated animals did not differ in comparison to animals that received SC only (*MO* + SC versus MC + SC, *p* > 0.05; HU + SC versus MC + SC, *p* > 0.05; RA + SC versus MC + SC, *p* > 0.05).

#### 4.2.2. Motor Coordination

A one-way ANOVA analysis revealed significant differences in motor coordination of rats expressed as their exit time from the cylinder (*F*(5,73) = 2.84, *p* < 0.05) (Table 2). Detailed analysis showed that the multiple administration of RA + H_2_O and HU + H_2_O did not affect significantly this paradigm when compared with control rats (*p* > 0.05), whereas* MO* treatment led to prolonged exit time (*MO* + H_2_O versus MC + H_2_O, *p* < 0.05). Moreover, generally SC-treated animals showed produced prolongation of exit time and the effects were statistically significant not only in control groups (MC + SC versus MC + H_2_O, *p* < 0.05), but also in RA-treated rats (RA + SC versus MC + SC, *p* < 0.05). However, the rest of the SC-treated animals did not differ in comparison to animals receiving SC only (*MO* + SC versus MC + SC, *p* > 0.05; HU + SC versus MC + SC, *p* > 0.05).

#### 4.2.3. Long-Term Memory

A one-way ANOVA analysis revealed significant differences in long-term memory after using a passive avoidance test (*F*(7,77) = 20.1; *p* < 0.05, Table 2). It was shown that the strongest effect leading to an improvement of this paradigm was produced by extract of* MO* and HU, but not RA, when compared with control animals (*MO* + H_2_O versus MC + H_2_O, *p* < 0.05; HU + H_2_O versus MC + H_2_O, *p* < 0.05; RA + H_2_O versus MC + H_2_O, *p* > 0.05), However, the administration of SC to rats significantly decreased the latency time of passive avoidance task (MC + SC versus MC + H_2_O, *p* < 0.05). After* MO* or RA combined treatment with SC, no improvement of long-term memory was observed, but HU given with SC showed enhancement of this paradigm in rats (HU + SC versus MC + SC, *p* < 0.05). Therefore, it can be concluded that administration of HU overcomes the effect shown by SC only (Table 1).

#### 4.2.4. Short-Term Memory

The results of the object recognition test showed that an administration of the compounds or extract did affect the rats' short-term memory (ANOVA *F*(7,78) = 2.73, *p* < 0.05) (Table 2).

Detailed* post hoc* analysis showed that only SC significantly decreased the short-term memory in* MO*-treated rats (*MO* + SC versus* MO* + H_2_O, *p* < 0.05;* MO* + SC versus MC + SC, *p* < 0.05), whereas the differences between the rest of the animals did not reach statistical significance (*p* > 0.05).

### 4.3. AChE and BuChE Activities in Rat Brain

A one-way ANOVA revealed significant differences between groups in the activity of AChE in both the cortex and the hippocampus (frontal cortex: *F*(3, 27) = 5.65, *p* < 0.05; hippocampus: ANOVA *F*(3,29) = 7.96, *p* < 0.05). It was found out that* MO* showed an insignificant inhibition of AChE activity in the frontal cortex by 24% when compared with control rats (MC + H_2_O, *p* < 0.06) and in the hippocampus by 7% (Table 3), whereas HU produced a distinct significant inhibition of AChE activity in comparison to control group by 48% (*p* < 0.05) and 47% (*p* < 0.05) in the cortex and the hippocampus, respectively. Also, RA lowered significantly AChE activity both in the cortex (38%) and in the hippocampus (43%). Moreover, there were not significant differences between the values of BuChE activities for* MO* and HU when compared with control group in the frontal cortex (ANOVA *F*(3,30) = 1.01, *p* > 0.05), whereas in the hippocampus the differences reached statistical significance (ANOVA *F*(3,27) = 14.1, *p* < 0.05). Detailed analysis showed that only RA effect was significant and increased BuChE activity in the hippocampus when compared with the control rats (*p* < 0.05).

### 4.4. AChE, BuChE, and BACE1 mRNA Level Changes in Rat Brain

A one-way ANOVA analysis revealed significant differences of AChE mRNA transcription profile in the cortex (ANOVA *F*(3,27) = 8.57, *p* < 0.05). As shown in Table 3, the multiple treatment of* MO* produced in the cortex a statistically significant decrease of AChE mRNA level by 52%; the administration of HU caused decrease of its level by 44% (*p* < 0.05), whereas RA did not affect this parameter when compared to the control.

There were significant differences between the relative values of BuChE mRNA levels in this region of brain in rats (frontal cortex: ANOVA *F*(3,25) = 16.2, *p* < 0.05). The* MO* treatment led to a decrease in the BuChE mRNA level by 84% (versus MC + H_2_O, *p* < 0.05), the prolonged HU administration resulted in a decrease of the transcript level in the cortex by 58% (versus MC + H_2_O, *p* < 0.05), but RA increased this parameter in the cortex by 84% (versus MC + H_2_O, *p* < 0.05).

The significant differences of mRNA transcription level of AChE mRNA level in the hippocampus (ANOVA *F*(3,31) = 21.2, *p* < 0.05) have been observed. The detailed analysis shown that, in the case of AChE after* MO* treatment, mRNA level significantly decreased by 69% (versus MC + H_2_O, *p* < 0.05), while the administration of HU resulted in a statistically insignificant decrease of AChE mRNA level by 18%, when compared with control group. Also RA did not change the level of transcript.

In the case of BuChE mRNA expression in the hippocampus, there were statistically significant differences between groups (ANOVA *F*(3,27) = 3.10; *p* < 0.05). Detailed analysis showed that, in the* MO* + H_2_O treated group, the expression lowered by 36%, but the difference did not reach strong significance when compared with the control values (*p* < 0.07) and no change in the expression level of this enzyme was shown after the administration of HU. On the contrary, RA produced an increase of BuChE mRNA expression in the hippocampus by 44% (versus MC + H_2_O, *p* < 0.05).

Further analysis showed the significant differences in BACE1 mRNA expression in both brain regions of rats (in the cortex and the hippocampus) (frontal cortex: ANOVA *F*(3,27) = 16.3, *p* < 0.05; hippocampus: ANOVA *F*(3,27) = 13.3, *p* > 0.05). It was observed that* MO* produced a statistically significant decrease of the BACE1 expression level by 64% in the cortex (versus MC + H_2_O, *p* < 0.05) and by 50% in the hippocampus (versus MC + H_2_O, *p* < 0.05). For comparison, HU treatment led to a decrease in the mRNA expression level by 38% in the cortex (versus MC + H_2_O, *p* < 0.05), but not in the hippocampus. On the contrary, RA produced an increase of this transcript in the cortex by 26%, but the effect did not reach a strong statistical significance (versus MC + H_2_O, *p* < 0.07), whereas in the hippocampus there was no difference between RA and control group.

## 5. Discussion

*Cognitive and Behavioral Experiments*. The present study investigated the influence of subchronic (28-fold) administration of standardized 50% EtOH extract of* Melisa officinalis* leaf extract (*MO*) (200 mg/kg, p.o., containing 17.7 mg/kg of RA) on SC-induced impairment of short-term and long-term memory in rats. The results were compared with the activity of cholinesterases (AChE and BuChE) as well as with AChE, BuChE, and BACE1 gene expression levels in the cortex and hippocampus of the rat brain. So far, little evidence is yet available as regards mechanisms of* MO* leaf extract action that are potentially relevant to cognitive function of rats after* per os* administration.* MO* is traditionally used in treating neurological disorders through its anti-AChE [18] and antiagitation properties [37]. Moreover, Wake et al. [38] and Kennedy et al. [15] showed that* MO* extract has nicotinic receptor activity and that it can displace [3H]-(*N*)-nicotine from nicotinic receptors in homogenates of human cerebral cortex tissue and they suggested that these mechanisms can explain activity of* MO* extract in amnesia model. Recently, Soodi et al. [18] observed that intraperitoneal injections of* MO* extract (200 mg/kg) in rats could significantly enhance learning and memory processes in animals since the extract significantly ameliorates SC-induced learning deficit in Morris water maze test. On the contrary, in higher dose, it can be observed that* MO* extract (400 mg/kg) could not reverse SC-induced memory impairment [18]. In our study, administration of* MO* extract at a dose of 200 mg/kg (p.o.) showed the effect leading to improvement of long-term memory in a passive avoidance test in rats. However, after* MO* combined treatment with SC,* MO* did not overcome the impairment shown by SC (Table 2). It should be emphasized that it is not clear whether the effect shown in our study by* MO* in non-SC-treated rats is specific, since the* MO* treatment produced significant lowering of locomotor activity of rats; therefore the sedative profile of* MO* cannot be excluded.

On the other hand, Ryu et al. [39] observed that agitation and aggression are highly prevalent in patients with dementia. According to Gitlin et al. [40], nonpharmacological interventions are recommended as first-line therapy. Although antipsychotics have shown benefit for Alzheimer's disease-related psychosis, their use is associated with several serious adverse effects [40]. Thus, it seems that the use of* MO* extract can provide dual benefits, both in aspect of inhibition of agitation and in improving the memory of patients with Alzheimer's disease.

Furthermore, in our study, RA in the dose of 10 mg/kg b.w. (p.o.) did not affect either short- or long-term memory, although RA lowered significantly AChE activity both in the cortex and in the hippocampus. Moreover, we observed that the repeated administration of RA in non-scopolamine-treated rats did not produce any changes of locomotor activity, similarly to our previous study [23].

It is possible that other chemical compounds can influence the memory in rats by synergic interactions in plant extract. According to our calculations, caffeic acid (0.174 mg in a single dose of extract administered to animals per kg b.w.), lithospermic acid (0.084 mg/kg), salvianolic acid A (0.08 mg/kg), and salvianolic acid B (0.046 mg/kg) may be responsible for observed pharmacological effects. On the other hand, results from few studies showed that salvianolic acids do not cross the blood brain barrier (BBB) [41, 42] and also lithospermic acid does not efficiently cross the BBB [43]. For this reason, the interpretation of our results is more complicated.

Firstly, Xu et al. [44] demonstrated that Sal A is a metabolically unstable compound that would undergo rapid methylation metabolism catalyzed by catechol O-methyltransferase* in vivo* into four major methylated metabolites of Sal A (3-O-methyl, 3′-O-methyl, 3,3′′-O-dimethyl, and 3′,3′′-O-dimethyl salvianolic acid A). These generated* O*-methylated metabolites may be largely responsible for its* in vivo* pharmacological effects. Although there are no available recent studies on the ability of these compounds to pass the BBB, such possibility should not be excluded.

Secondly, several studies showed central pharmacological effects in animals after* per os* administration of Sal B [45, 46], Sal A [47], and caffeic acid [48]. It was proved, for example, that salvianolic acid B (10 mg/kg, p.o.) significantly rescued the A*β*25–35 peptide-induced decrease of choline acetyltransferase and brain-derived neurotrophic factor protein levels in an amyloid *β* (A*β*) peptide-induced Alzheimer's disease mouse model [49]. It also significantly reversed (10 mg/kg, p.o.) the cognitive impairments induced by scopolamine (1 mg/kg, i.p.) or A*β* (25–35) (10 nmol/5 *μ*L, i.c.v.) injection in mice [45]. Previous studies [46, 47] showed also that both Sal A and Sal B are able to improve the impaired memory function induced by cerebral ischemia-reperfusion in mice. The stimulation of neurogenesis process in both subgranular zone (SGZ) and subventricular zone (SVZ) after brain ischemia and also alleviation neural cells loss and improved motor function recovery after brain ischemia in rats after the Sal B administration were also observed by Zhong et al. [50]. Moreover, the exposure to Sal B can maintain the proliferation of neural stem/progenitor cells (NSPCs) after cerebral ischemia and improve cognitive postischemic impairment after stroke in rats using Morris water maze test; therefore authors concluded that Sal B may act as a potential drug in treatment of brain injury or neurodegenerative disease [51]. Additionally, the improvement of motor function after cerebral ischemia in rats after salvianolic acid B administration was also demonstrated [52]. The clue to pass through the BBB Sal B provided also results of Li et al. indicating its protective effect on BBB in rats after cerebral ischemia-reperfusion by inhibiting the MAPK pathway [53]. In addition to this, it was shown that lithospermic acid and salvianolic acids exerted neuroprotective activity in various experimental models. Lithospermic acid significantly attenuates neurotoxicity* in vitro* and* in vivo* induced by 1-methyl-4-phenylpyridin (MPP(+)) by blocking neuronal apoptotic and neuroinflammatory pathways [54]. Salvianolic acid B inhibited amyloid beta-protein aggregation and fibril formation, as well as directly inhibiting the cellular toxicity of amyloid beta-protein in PC12 cells [55], and significantly reduced its cytotoxic effects on human neuroblastoma SH-SY5Y cells [56].

For an explanation of our results, observations of Pinheiro Fernandes et al. [57] may be very helpful, which showed that caffeic acid, nonflavanoid catecholic compound, whose derivatives are occurring in* MO* extract, improved the working, spatial, and long-term aversive memory deficits induced by focal cerebral ischemia in mice. Anwar et al. [48] showed also that caffeic acid (100 mg/kg) improved the step-down latencies in the inhibitory avoidance in rats. Tsai et al. [58] showed that caffeic acid is a potent neuroprotective agent in brain of 1-methyl-4-phenyl-1,2,3,6-tetrahydropyridine (MPTP) treated mice. Moreover, caffeic acid improves A*β*25–35-induced memory deficits and cognitive impairment in mice [59]. In another study, it was also shown that this compound has a significant protective effect on global cerebral ischemia-reperfusion injury in rats [60]. For instance, 12.4 ± 1.8 mg/100 g of caffeic acid was detected in the brain of mice with a diet containing 2% caffeic acid for 4 weeks [58]. Thus, there is progressive evidence that this compound passes through the BBB and has a central pharmacological activity.

Also results by Yoo et al. are also worth noting [61] which showed, also in a rat model of scopolamine-induced amnesia, that administration of luteolin (a common flavonoid from many plants including* M. officinalis*) at dose of 10 mg/kg caused the increase in the brain-derived neurotrophic factor (BDNF), acetylcholine, and the decrease in lipid peroxidation. Liu et al. [62] observed that chronic treatment with luteolin (50 and 100 mg/kg) improved neuronal injury and cognitive performance by attenuating oxidative stress and cholinesterase activity in streptozotocin-induced diabetes in rats. In our study, ethanolic extract of* MO* administered to rats significantly improved long-term memory after using a passive avoidance test, but after* MO* combined treatment with SC no improvement of this paradigm was observed (Table 2).

Thirdly, available pharmacokinetic studies were carried out for the pure compounds (Sal A and B) and the extract of the roots of* Salvia miltiorrhiza* [42, 63], but there are not available studies of bioavailability of these compounds after administration of* Melissa officinalis* leaf extract, containing other compounds as compared with the extract of* Salvia miltiorrhiza*. Moreover, there is a lack of data about how scopolamine may influence the penetration of the salvianolic acids (and other compounds) across the BBB. Hence, there is a need to study the pharmacokinetic parameters of these compounds in the group of animals treated with scopolamine in comparison with control group.

*AChE and BuChE Activities in Rat Brain*. To date, several studies have focused on explaining the mechanism of action of the MS extract and its active compounds. Soodi et al. [18] showed that treatment of animals with* MO* extract (400 mg/kg) prior to scopolamine injection could ameliorate scopolamine-induced enhancement in AChE activity. This dose of* MO* extract inhibited the AChE activity in the hippocampus of rats (51.9% versus 100.4% in normal saline group, *p* < 0.05; 91.4% in group treated with scopolamine +* MO* versus 128.1% in scopolamine group, *p* < 0.05). In another study, Anwar et al. [48] showed that 50 and 100 mg/kg of caffeic acid decreased* in vivo* the AChE activity in the cerebral cortex and striatum and increased the activity of this enzyme in the cerebellum, hippocampus, hypothalamus, pons, lymphocytes, and muscles when compared to the control group (*p* < 0.05). Our study showed that ethanolic extract of* MO* produced an insignificant and slight inhibition of AChE and BuChE activity in the frontal cortex and in the hippocampus (especially). However, it was observed previously [23] that RA inhibited the AChE activity in the rats frontal cortex and hippocampus. Moreover, we showed that RA possess a strong stimulatory effect on BuChE in the hippocampus.

*AChE, BuChE, and BACE1 mRNA Level Changes in Rat Brain*. So far, no results have been published of studies concerning the* in vivo* assessment of changes in AChE, BuChE, and BACE1 gene expression profile in different brain regions under the influence of* MO* extracts or their key bioactive metabolites. Such studies focused overwhelmingly on the analysis of their* in vitro* activities, to a lesser extent in animal experiments.

In our study, in the frontal cortex, we have observed the strongest inhibition of both AChE and BuCHE mRNA transcription under the influence of* M. officinalis* extract and huperzine A (Table 3). However, a more significant difference in the level of transcript was seen in the frontal cortex, in particular in the case of BuCHE mRNA of experimental rats (a decrease by 51.6% and 44% for* MO* and HU versus control, resp.). The strong inhibition of transcription of AChE mRNA was also observed in the hippocampus of* MO*-treated rats (a decrease by 69%). The BuChE mRNA transcription in animals receiving* MO* was lowered in a moderate way (decrease by 36%). Huperzine A alone has not caused changes that were observed in the group of* MO* (Table 2). Our findings mostly correlate with the observed changes in activities of AChE and BuChE in different groups of animals. In the case of BuChE, slightly different results between its activity and expression profile were especially seen in the frontal cortex and hippocampus of animals receiving* MO* (Table 3).

To date, there is a lack of published results of studies making an attempt to clarify the potential differences in the transcriptional profile and activity of AChE and BuChE under the influence of* MO* and its bioactive metabolites. It cannot be excluded that the key for the observed differences in the level of AChE activities and mRNA level can be caused by changes in the activity of AChE in other regions of the brain, not analyzed in this study, such as substantia nigra, cerebellum, globus pallidus, and hypothalamus, where it exerts nonenzymatic neuromodulatory functions affecting neurite outgrowth and synaptogenesis, modulating the activity of other proteins regional cerebral blood flow, and other functions [64]. But it is difficult to clearly explain why diminishing of AChE and BuChE mRNAs did not always correlate with the lowering of activity of these enzymes, although it has been recently noted that AChE activity was not paralleled by an increase in mRNA levels [65]. The authors explained this fact by stating that AChE levels are regulated at transcriptional, posttranscriptional, and posttranslational levels leading to complex expression patterns which can be modulated by physiological and pathological conditions. However, these mechanisms are not fully understood and further studies are needed in this field.

The cause of observed different degree of inhibition of activity and transcription status of studied genes, especially of BuChE (and AChE), may lie in a so-called “negative feedback” consisting of a complicated transcription/translation regulation, protein-protein interactions/modifications, and a metabolic network, together forming a system that allows the cell to respond sensibly to the multiple signal molecules that exist in its environment [66].

Because of that, we propose that the reason for differences between AChE and BuChE activities in the cortex and hippocampus under the* MO* may be due to the fact of insufficiency of applied dose and the duration of the experiment, affecting the transcriptional, tissue-specific, cellular machinery regulating BuChE transcription without affecting its activity. Moreover, the administration of higher doses of* MO* and extended period of time could lead to sufficient inhibition of its activity.

There is a need to conduct further studies to determine the molecular degree of dependence between changes in AChE and BuChE activities and activities of potential key factors regulating expression of these genes in the frontal cortex and hippocampus under the influence of extracts of* MO* and active metabolites. Another study determining the effectiveness of their actions on the cholinergic system in experimental animal models of memory impairment, with particular emphasis on scopolamine, including the determination of changes at the molecular level should be therefore carried out.

Alterations in BACE1 protein level have been proved in postmortem brain tissue from individuals with AD, with increases, decreases, and also no change reported [1, 67, 68]. An example of confirmation of these findings at the mRNA level is results of study by Coulson et al. [69]. Based on conducted studies, some authors suggested that increased BACE1 mRNA transcription in remaining neuronal cells may contribute to the increased BACE1 protein levels and activity found in brain regions affected by AD [70].

Results of many studies concerning the elevated level of BACE1 mRNA and protein in Alzheimer's disease provide direct and compelling reasons to develop therapies directed at BACE1 inhibition, thus reducing *β*-amyloid and its associated toxicities [67, 68].

In our study, we have observed that BACE mRNA expression statistically significantly decreased after* MO* administration in frontal cortex and hippocampus. These results suggest that the* MO* extract may act to inhibit BACE1 mRNA level, given the fact that the percentage of inhibition of the expression (64% in the frontal cortex, 50% in the hippocampus) is higher than that in the case of HU (38% in the cortex and the lack of changes in the hippocampus). A careful analysis of the literature data shows that there are no studies which analyzed the impact of* MO* extract on the expression level of BACE1 in Alzheimer's disease.

Furthermore, a literature analysis does not indicate already published results conducted by other teams attempting to assess the impact of RA on the transcriptional activity of AChE, BuChE, and BACE1. Although several studies (already mentioned and others [70, 71]) highlighted the RA and other caffeic acid derivatives capability of acetylcholinesterases inhibition, none of these studies does not touch the question of the molecular basis of their impact on the transcriptional machinery that regulates* in vivo* the expression of studied genes. Hence, in our opinion, obtained by our team results, they are one of the first of this type and, in general, correlate with the results of our previous study [23]. In this case, there is no clear evidence explaining different responses at the transcriptional level under the influence of RA, especially in the case of BuChE encoding gene in the hippocampus (Table 2). It is possible that the observed differentiation of BuChE transcriptional activity between the frontal cortex and the hippocampus may be due to the differences of butyrylcholinesterase localization and substrate affinity [72]. Since in our experiment we have carried out a quantitative analysis of AChE, BuChE, and BACE1 transcripts in brain homogenates of tested animals, rather than in individual, isolated cell fractions, therefore the obtained results constitute an overall “picture” of both studied genes transcriptional changes occurring in the brain areas of studied animals under the influence of the RA and the whole plant extract as well.

## 6. Conclusion

The subchronic administration of* MO* led to an improvement of long-term memory of rats; however the mechanisms of* MO* action are probably more complicated, since its role as a modulator of beta-secretase activity (due to inhibition of BACE1 mRNA expression in frontal cortex) should be taken into consideration.

It should be noted that we have studied a crude extract from leaf of* Melissa officinalis*, not a single pure chemical compound. This plant extract is a complex mixture, and its action may be a result of the summation of activities of several components (synergism/additive action of caffeic acid with salvianolic acids, rosmarinic acid, and others). In the case of extract from leaves of* Melissa officinalis*, it is possible that interactions occur between the 40 chemical compounds identified by HPLC system.

Taken together, it seems that the* MO* activity represents a possible option as complementary interventions to relieve the symptoms of mild dementia.

## Figures and Tables

**Table 1:** Metabolites detected in *Melissa officinalis* leaf extract by UPLC-MS.

No	RT [min]	Metabolite identification	Chemical formula	Exact mass of [M-H]^−^		Δppm	Fragmentation in		*λ*_max_ [nm]	CID^a^	Identification level^b^	Reference
				Measured	Calculated		Negative ion mode (ESI−)	Positive ion mode (ESI+)				
1	1.41	2-Hydroxy-3-(3,4-dihydroxyphenyl)-propanoic acid	C_9_H_9_O_5_	197.045	197.0455	−4.1775	197, 179, 135	199, 163	283, 312	8143997	2	[34]

2	1.73	Dihydroxybenzoic acid hexoside	C_13_H_15_O_9_	315.072	315.0722	0.8346	315, 153, 109	317, 155	282	54726828	3	[35]

3	1.98	Caftaric acid	C_13_H_11_O_9_	311.041	311.0409	0.6141	311, 221, 179, 149		318	6440397	2	[35]

4	2.24	2-Hydroxy-3-(3,4-dihydroxyphenyl)-propanoic acid sulphated	C_9_H_9_O_8_S	277.003	277.0024	0.3998	277, 197, 179, 135		312		3	[35]

5	2.56	Hydroxyjasmonic acid hexoside	C_18_H_27_O_9_	387.166	387.1661	0.9214	387, 207, 163		323	44237366	2	[17]

6	2.76	Caffeic acid	C_9_H_7_O_4_	179.034	179.0451	−4.9075	179, 135	181, 163	275	689043	1	std

7	2.89	Salvianolic acid E	C_36_H_29_O_16_	717.1450	717.1467	2,313	717, 519, 339, 321, 295, 277		275, 325	49770697	2	[35]

8	3.17	Salvianolic acid H/I (isomer)	C_27_H_21_O_12_	537.104	537.1038	0.8562	537, 493, 359, 295		281, 325		2	[35]

9	3.45	Hydroxyjasmonic acid sulphated	C_12_H_17_O_7_S	305.07	305.07	1.117	305, 225, 194, 147		275, 330		3	[17]

10	3.62	Nepetoidin B	C_17_H_13_O_6_	313.072	313.0718	1.1232	nd		282, 316	5316819	2	[73]

11	3.7	Yunnaneic acid F	C_26_H_25_O_14_	597.1255	597.1244	1.8743	597, 509, 311, 197		Masked		2	[34]

12	3.83	Decarboxyrosmarinic acid (teucrol)	C_17_H_15_O_6_	315.088	315.0874	0.9396	315, 179, 135		275, 330	637829	2	[74]

13	3.9	Caffeoylcaftaric acid	C_22_H_17_O_12_	473.073	473.0725	0.7555	173, 311, 149		Masked	65018	3	[35]

14	3.97	Apigenin glucosylrhamnoside	C_27_H_29_O_14_	577.157	577.1563	1.1136	577, 269	579, 433, 271	275, 340	92741003	3	[33]

15	4.04	Luteolin 7-*O*-glucoside 3′-*O*-glucuronide	C_27_H_27_O_17_	623.126	623.1254	0.9696	623, 461, 447, 285, 255	625, 463, 287	273, 343		2	[33]

16	4.21	Rosmarinic acid hexoside	C_24_H_25_O_13_	521.13	521.1301	0.3551	521, 359, 161	523, 361, 325, 163	329	25245848	2	[34]

17	4.3	Luteolin *O*-diglucoside	C_27_H_29_O_16_	609.147	609.1461	0.7183	609, 285	611, 287	269, 349		3	[33]

18	4.41	Luteolin glucosylrhamnoside	C_27_H_29_O_15_	593.152	593.1512	0.8077	593, 447, 285	595, 449, 287	271, 343		3	[33]

19	4.49	Luteolin 4′-*O*-glucoside	C_21_H_19_O_11_	447.094	447.0933	0.9728	447, 285	449, 287	271, 343	5319116	3	[33]

20	4.5	Sagerinic acid 2-hydroxy-3-(3,4-dihydroxyphenyl)-propanoide	C_45_H_39_O_20_	899.205	899.204	1.0448	899, 719, 591, 475, 295		Masked		3	[35]

21	4.9	Salvianolic acid B (lithospermic acid B)	C_36_H_29_O_16_	717.146	717.1461	0.1846	717, 519, 359, 161		327	6441188	2	[34]

22	5.04	Sagerinic acid	C_36_H_31_O_16_	719.163	719.1618	0.9979	719, 519, 359, 161		287, 330		2	[34]

23	5.14	Rosmarinic acid	C_18_H_15_O_8_	359.077	359.0772	0.002	359, 161	361, 163	329	5281792	1	std

24	5.55	Salvianolic acid B 2-hydroxy-3-(3,4-dihydroxyphenyl)-propanoide	C_45_H_37_O_20_	897.19	897.1884	1.6873	897, 717, 519, 359, 161		330		3	[35]

25	5.6	Sagerinic acid di-2-hydroxy-3-(3,4-dihydroxyphenyl)-propanoide	C_54_H_47_O_24_	1079.2449	1079.2457	−0.7911	1079, 897, 719, 539, 359, 295		288, 330		3	[35]

26	5.92	Sagecoumarin di-2-hydroxy-3-(3,4-dihydroxyphenyl)-propanoide caffeide	C_54_H_43_O_24_	1075.2156	1075.2150	0.559	1077, 897, 717, 537, 409, 359, 339, 277		322		3	[35]

27	5.98	Rosmarinic acid sulphated I isomer	C_18_H_15_O_11_S	439.035	439.0341	2.0214	439, 359, 341, 163		Masked		2	[34]

28	6.13	Luteolin 3′-*O*-glucuronide	C_21_H_17_O_12_	461.073	461.072	1.634	461, 285	463, 287	269, 340	170474237	2	[33]

29	6.32	Salvianolic acid A	C_26_H_21_O_10_	493.115	493.114	1.1011	493, 359, 295, 179		298, 327	5281793		[34]

30	6.56	Sagerinic acid sulphated	C_36_H_31_O_19_S	799.1196	799.1186	0.954	799, 719, 619, 519, 359, 161		325		3	[35]

31	6.71	Lithospermic acid	C_27_H_21_O_12_	537.104	537.1048	0.9698	537, 493, 359, 161		292, 329	6441498	2	[35]

32	6.8	Rosmarinic acid sulphated II isomer	C_18_H_15_O_11_S	439.034	439.0341	0.2837	439, 359, 341, 163		Masked		2	[34]

33	6.89	Sagecoumarin	C_27_H_19_O_12_	535.088	535.0882	0.1204	535, 311, 267, 177		Masked		2	[36]

34	7.03	Salvianolic acid L I isomer	C_36_H_29_O_16_	717.146	717.1461	0.2697	717, 519, 359		284, 329		2	[35]

35	7.1	Salvianolic acid L hydroxycaffeide	C_45_H_35_O_20_	895.173	895.1727	0.6282	895, 519, 359, 161		Masked		3	[35]

36	7.4	Sagecoumarin caftaride	C_40_H_29_O_20_	829.126	829.1258	0.6953	829, 667, 535, 355, 311		Masked		3	[36]

37	7.51	Sagecoumarin 2-hydroxy-3-(3,4-dihydroxyphenyl)-propanoide	C_36_H_27_O_16_	715.131	715.1305	0.8176	715, 535, 311, 267		319		3	[34]

38	7.71	Unknown	C_36_H_57_O_14_S	745.348	745.3475	0.4402			281, 326		4	[35]

39	7.85	Methyl rosmarinate	C_19_H_17_O_8_	373.093	373.0929	0.1765	373, 359, 161		284, 323	6479915	2	[75]

40	8.69	Salvianolic acid C caffeoylhydroxycaffeide	C_44_H_33_O_18_	849.168	849.1672	0.8615	849, 687, 491, 359, 327, 255		286, 318		3	[35]

^a^CID: identifier for a chemical structure in the PubChem Compound database.

^b^Metabolite identification level according to Metabolomics Standards Initiative recommendation [76].

std: identification on the basis of standard compound fragmentation.

nd: not detected.

**Table 2:** Effect of *Melissa officinalis* leaf extract (200 mg/kg, p.o.) treatment on sedative activity, motor coordination, and memory in rats.

Group	*n*	Locomotor activity [number of impulses/5 min]	Motor coordination, exit time [s]	Short-term memory^e^ OR	Long-term memory, latency [s]
MC + H_2_O	18	390 ± 24	17 ± 3	0.40 ± 0.06	47 ± 14
MC + SC	18	526 ± 48^*∗*^	32 ± 5^*∗*^	0.32 ± 0.05	12 ± 3^*∗*^
*MO* + H_2_O	10	231 ± 48^*∗*^	32 ± 7^*∗*^	0.43 ± 0.07	169 ± 11^*∗*^
*MO* + SC	9	436 ± 60	43 ± 7	0.09 ± 0.11^*∗*^	23 ± 6
HU + H_2_O	9	515 ± 32	15 ± 2	0.37 ± 0.09	158 ± 14^*∗*^
HU + SC	8	639 ± 71^*∗*^	56 ± 3	0.22 ± 0.06	49 ± 18^#^
RA + H_2_O	8	406 ± 59	21 ± 5	0.45 ± 0.05	58 ± 28
RA + SC	8	605 ± 55	28 ± 7	0.45 ± 0.05	20 ± 7

Means ± SEM.

^*n*^Number of animals.

MC + H_2_O: control rats.

SC: scopolamine (0.5 mg/kg b.w., i.p.).

HU: huperzine A (0.5 mg/kg b.w., p.o.).

RA: rosmarinic acid (10 mg/kg b.w., p.o.).

^e^Expressed as ratio OR = (*B* − *A* ^*∗*^)/(*B* + *A*); for details see Section 3.

^*∗*^Versus MC + H_2_O, *p* < 0.05.

^#^Versus MC + SC, *p* < 0.05.

**Table 3:** The effect of *Melissa officinalis* leaf extract on acetylcholinesterase (AChE) and butyrylcholinesterase (BuChE) activities and AChE, BuAChE, or beta-secretase (BACE1) mRNA expression levels in frontal cortex (FC) or hippocampus (Hipp) of rats.

Group^*n*^	Enzyme activity [nmol/min/mg protein]				mRNA expression^#^ [%]					
	AChE		BuChE		ACHE		BuChE		BACE1	
	FC	Hipp	FC	Hipp	FC	Hipp	FC	Hipp	FC	Hipp
MC + H_2_O	363 ± 49	439 ± 73	65 ± 11	53 ± 8	100 ± 12	100 ± 11	100 ± 18	100 ± 11	100 ± 16	100 ± 8
*MO* + H_2_O	276 ± 34	409 ± 28	69 ± 6	62 ± 4	48 ± 4^*∗*^	31 ± 7^*∗*^	16 ± 2^*∗*^	64 ± 21^&^	36 ± 3^*∗*^	50 ± 8^*∗*^
HU + H_2_O	189 ± 15^*∗*^	239 ± 15^*∗*^	58 ± 6	51 ± 4	53 ± 13^*∗*^	85 ± 5	42 ± 9^*∗*^	102 ± 11	62 ± 6^*∗*^	98 ± 4
RA + H_2_O	224 ± 16^*∗*^	251 ± 12^*∗*^	77 ± 7	99 ± 7^*∗*^	101 ± 12	103 ± 5	184 ± 31^*∗*^	56 ± 7^*∗*^	126 ± 13^&^	98 ± 3

Means ± SEM.

^*n*^Number of animals: 7–10.

^#^Values expressed as a ratio: the gene/GAPDH.

MC + H_2_O: control group.

HU: huperzine A (0.5 mg/kg b.w., p.o.).

RA: rosmarinic acid (10 mg/kg b.w., p.o.).

^*∗*,&^Versus MC + H_2_O, *p* < 0.05 or *p* < 0.07, respectively.
